# Spindle Cell Lipoma in the Lower Lip: A Report of a Rare Case in Plastic Surgery

**DOI:** 10.7759/cureus.77956

**Published:** 2025-01-25

**Authors:** Isao Nagata, Toshihito Mitsui, Yuki Matsuoka, Masakatsu Hihara, Natsuko Kakudo

**Affiliations:** 1 Department of Plastic and Reconstructive Surgery, Kansai Medical University, Hirakata, JPN

**Keywords:** benign tumor, lip, lipoma, spindle cell lipoma, surgical management

## Abstract

Spindle cell lipoma (SCL) is a rare subtype of lipoma. SCL is commonly reported in subcutaneous tissues of the neck, shoulders, and back, but its occurrence in the lip, where adipose tissue is sparse, is rare. We present a case of SCL in a 53-year-old woman referred to our plastic surgery department with a chief complaint of a submucosal mass in the right lower lip. Magnetic resonance imaging (MRI) revealed a mass in the submucosal tissue of the lower lip. Surgical excision was performed through a lip incision. Histopathological examination confirmed the diagnosis of SCL with the presence of CD34-positive/S100-negative cells. Differential diagnosis between SCL and liposarcoma is crucial and challenging based on clinical or imaging findings, emphasizing the importance of histopathological examination.

## Introduction

Lipomas are benign mesenchymal tumors composed of fibroadipose tissue. According to the WHO classification of benign adipocytic tumors, lipomas are categorized into several subtypes, including lipoma, lipomatosis, neurolipomatosis, lipoblastoma, lipoblastomatosis, angiolipoma, myolipoma, chondrolipoma, extrarenal angiomyolipoma, extra-adrenal myelolipoma, spindle cell lipoma (SCL), pleomorphic lipoma and hibernoma [1,2]. SCL, first described by Enzinger and Harvey in 1975, typically consists of mature adipocytes and spindle cells [3]. SCLs commonly arise as a solitary subcutaneous and well-delineated mass forming lesions of the posterior neck, shoulders, and back of males aged 40-70 years [4,5]. SCLs generally grow slowly, composed of mature adipose tissue surrounded by fibrous capsules. Lipomas represent 0.5-5% of all benign oral neoplasm [2], and oral SCLs are extremely rare, comprising only 2.2-9.8% of all oral lipomas [6,7]. Since SCLs have various characteristics, it is often difficult to identify the disease to distinguish it from other spindle cell neoplasms including lipomatous and non-lipomatous [8].

This report details a rare case of SCL arising in the submucosal tissue of the lower lip, along with its diagnosis and surgical management.

## Case presentation

**Figure 1:**
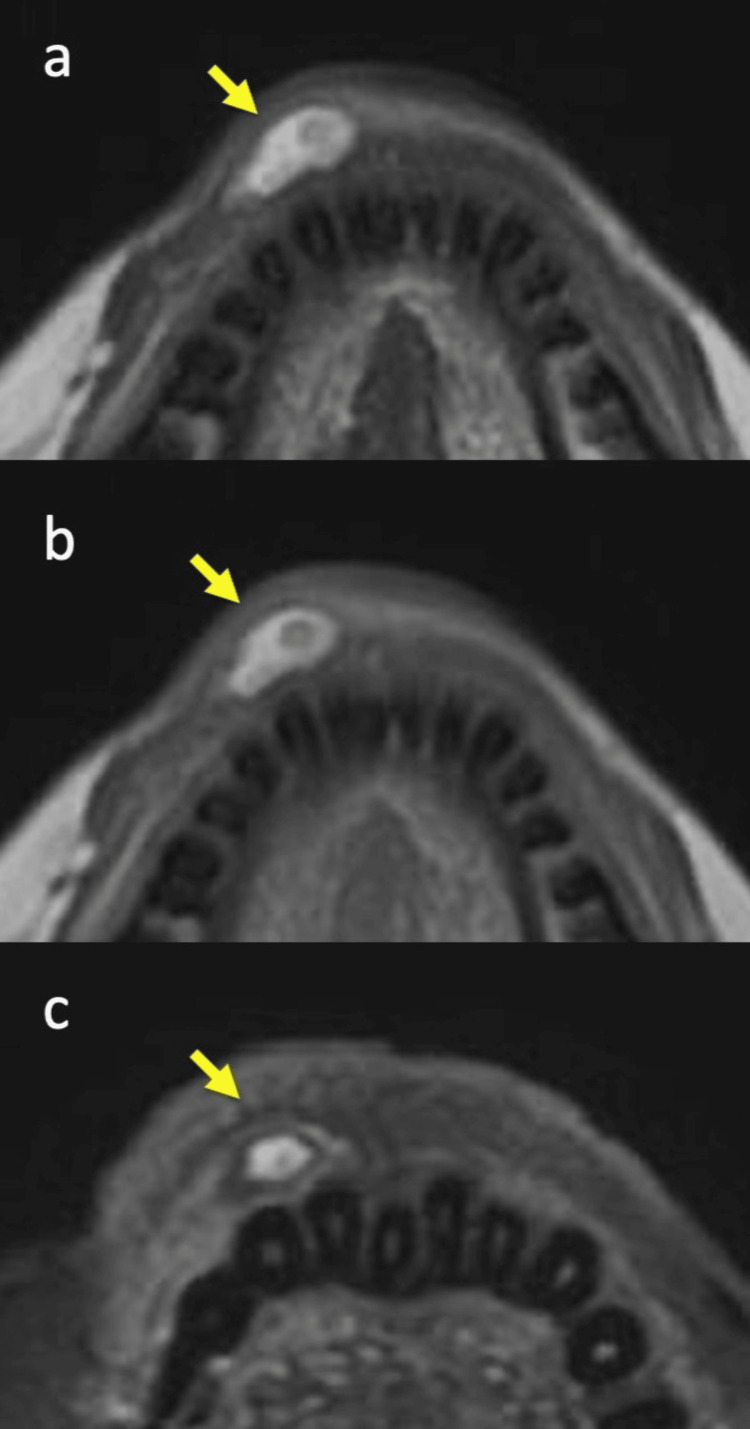
Magnetic resonance imaging (MRI) of the lower lip showing the tumor’s position (indicated by arrows). (a) T1-weighted axial image; (b) T2-weighted axial image; (C) Fat-suppressed axial image by short tau inversion recovery (STIR).

A 53-year-old woman presented to the dental and oral surgery department of our hospital with a chief complaint of a submucosal lump in the right lower lip. She was referred to our department due to suspicion of lipoma or liposarcoma based on MRI findings. Palpation revealed a soft and elastic mass. The patient reported no pain or other symptoms, apart from a sensation of swelling. She had noticed the mass six months prior, with no change in size since its discovery. Her medical and family histories were unremarkable, and no prior illnesses or allergies were reported. MRI demonstrated a relatively well-demarcated mass beneath the mucosa of the right lower lip, showing heterogeneously low-to-high signals on both T1- and T2-weighted images (Figures 1a, 1b). The fat-suppressed image by short tau inversion recovery (STIR) revealed that an adipose tissue-predominant nodule occupied the affected area (Figure 1c). Differential diagnoses included mucocele, liposarcoma, and myxoma, based on the lesion's characteristics.

**Figure 2:**
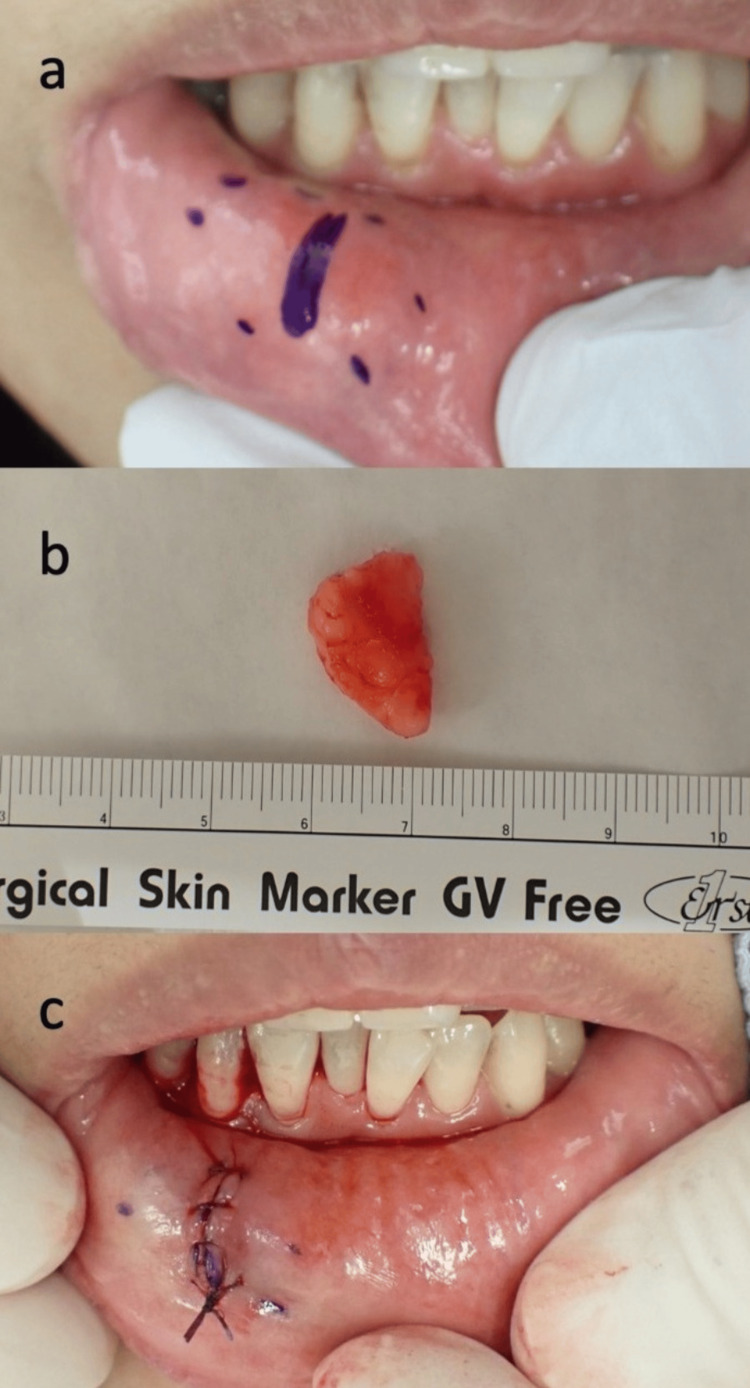
Surgical findings. (a) Spindle cell lipoma (SCL) located at the right lower lip; (b) Excised mass from the submucosa; (c) Postoperative appearance of the incision site.

Vital signs, including body temperature, blood pressure, and oxygen saturation, were within normal limits. Surgical excision was performed under local anesthesia. A vertical incision was made directly over the tumor, and the lesion was carefully removed above the orbicularis oris muscle (Figure 2a). The excised tumor measured approximately 17 × 14 mm, was yellowish, and exhibited elastic softness (Figure 2b). Given the exposed location, meticulous dissection was undertaken to ensure an aesthetically favorable postoperative outcome (Figure 2c).

**Figure 3:**
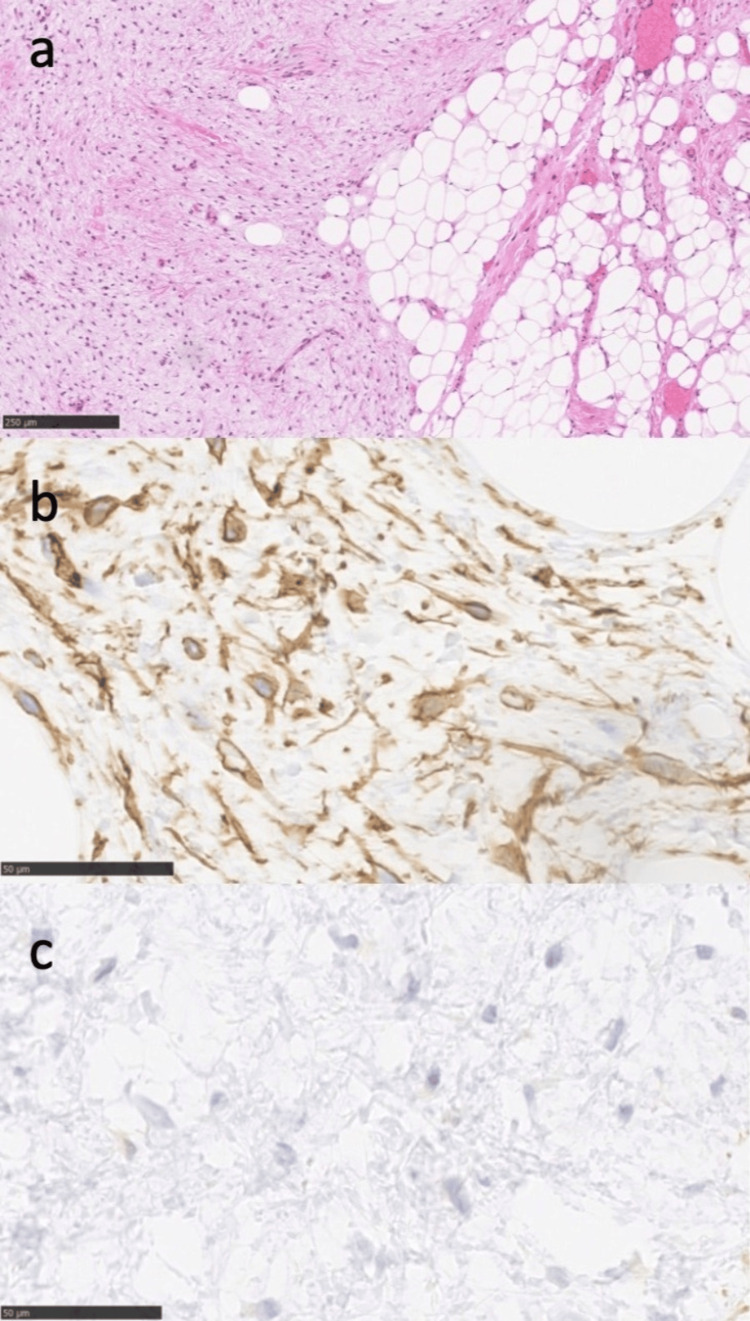
Histologic findings of spindle cell lipoma (SCL). (a) Hematoxylin and eosin staining. Scale bar: 250 μm; (b) Immunostaining showing a positive result for CD34. Scale bar: 50 μm; (C) Immunostaining showing a negative result for S100. Scale bar: 50 μm.

Histopathological examination with hematoxylin and eosin (HE) staining revealed the proliferation of mature adipocytes and spindle-shaped fibroblast-like cells within a myxoid stroma. Nuclear pleomorphism and mitotic figures were absent (Figure 3a). Immunohistochemical staining showed CD34 positivity in both adipocytes and spindle-shaped cells (Figure 3b), while S100-staining of spindle cells was negative (Figure 3c). These findings confirmed the diagnosis of SCL.

At six months postoperatively, there was no evidence of recurrence or abnormalities. The patient experienced no discomfort during speech or other activities.

## Discussion

SCL, first described by Enzinger and Harvey in 1975, accounts for approximately 1.5% of all lipomas [3]. This tumor predominantly occurs in the subcutaneous tissue of the neck, shoulders, and back, presenting as a painless, slowly growing, spherical or lobulated mass with well-defined borders. Among 114 reported cases, 104 occurred in males, with an age range of 25 to 75 years and an average age of 56 years [3]. SCL typically presents as a solitary lesion; however, reports indicate that 0.5% to 3% of cases exhibit multiple lesions [7]. McDaniel et al. documented the first case of SCL occurring in the oral cavity in 1984 [6]. While less than 10% of lipomas arise in the oral region, SCL constitutes only a small subset of these cases [2,6-8].

In the oral cavity, SCL predominantly affects the tongue (37%), buccal mucosa (31%), and, less frequently, the floor of the mouth (5%), with occasional occurrences in the hard palate and gingiva [9]. It is exceptionally rare for SCL to arise in the lips, where adipose tissue is absent [9]. Furlong et al. reported 12 cases of SCL involving the lips but provided no detailed descriptions of the patients’ symptoms or surgical procedures [10]. Our literature review identified only seven additional cases of SCL diagnosed in the lips [11]. Among Japanese patients, Saito and Watanabe documented a single case of a 13 mm SCL on the upper lip in a 57-year-old female [12]. Therefore, this case represents a rare diagnosis and surgical treatment of SCL on the lip, managed by a plastic surgeon.

The differential diagnosis of oral SCL includes salivary gland tumors, benign mesenchymal neoplasms, mucous extravasation phenomena, and mucoceles [13]. Histologically, SCL comprises CD34-positive spindle-shaped mesenchymal cells intermixed with mature adipocytes in varying proportions. The spindle cell areas are rich in acidic mucopolysaccharides, rope-like collagen fibers, and mast cells. Accurate differential diagnosis is critical as treatment and prognosis differ significantly from those of liposarcoma. Clinically, while lipomas typically arise in superficial subcutaneous adipose tissue, liposarcomas tend to occur in deeper tissues. However, physical examination findings of both affected areas are similar, and rare cases of liposarcoma in the lips have been reported [14].

Radiologically, both lipomas and liposarcomas display well-demarcated lesions with signal intensities equivalent to adipose tissue on CT and MRI, making imaging-based differentiation challenging [5,8]. Histopathologically, liposarcomas exhibit atypical spindle-shaped cells and lipoblasts, whereas these features are absent in SCL, making histopathology crucial for diagnosis. SCL exhibits diverse histological patterns due to the variable proportions of its components. Previously, distinguishing between well-differentiated liposarcoma and SCL was difficult [15]. Immunohistochemical findings have since facilitated definitive diagnosis; SCL consistently expresses CD34, while S100, expressed in liposarcomas, is negative in SCL [15,16]. In the present case, the lesion was confirmed as SCL based on CD34 positivity and S100 negativity (Figure 3). Additionally, immunohistochemical findings, including the loss of RB1 protein expression and the normal expression of MDM2 and CDK4, contribute to an accurate diagnosis [15].

SCL exhibits a spectrum of histological patterns, ranging from lesions dominated by mature adipocytes with scattered spindle cells to those predominantly composed of spindle cells with minimal adipocytes. In this case, the tumor consisted of a roughly equal ratio of spindle cells to adipocytes, representing a standard subtype. Due to the relatively small tumor size, surgical excision was straightforward. Previous reports have noted instances where biopsy specimens predominantly comprising spindle cells were misdiagnosed as solitary fibrous tumors, whereas adipocyte-rich areas led to a diagnosis of lipoma [15]. Comprehensive histopathological examination of the entire tumor is thus essential for an accurate diagnosis of SCL.

The etiology and pathogenesis of SCL remain unclear. However, persistent trauma, genetic predisposition, hormonal imbalances, and metabolic abnormalities are thought to contribute to its development [8]. The higher incidence in older males, with a male-to-female ratio of 1.9:1, suggests a potential role of male steroid hormones in SCL pathogenesis [4,5], although the exact mechanisms are unknown. The standard treatment for SCL is simple surgical excision. The prognosis following complete excision is generally favorable, comparable with that of conventional lipomas. However, some cases of recurrence have been reported, underscoring the importance of careful postoperative management and clear surgical resection margins.

## Conclusions

This report presents a rare case of SCL located in the lower lip. SCL is a benign tumor characterized by slow growth and an asymptomatic clinical course. Differentiation between SCL and liposarcoma is critical; however, imaging studies alone are often insufficient to establish a definitive diagnosis. A comprehensive approach that integrates physical examination findings and pathological evaluation is essential for accurate diagnosis. Given the relatively low-risk nature of SCL, surgical excision remains the standard treatment, with an excellent prognosis in the majority of cases.
